# Development of a Multiplexed Microfluidic Platform for the Automated Cultivation of Embryonic Stem Cells

**DOI:** 10.1177/2211068213499917

**Published:** 2013-12

**Authors:** Marcel Reichen, Farlan Singh Veraitch, Nicolas Szita

**Affiliations:** 1 Department of Biochemical Engineering, University College London, London, UK; * Current affiliation: Department of Haematology, University of Cambridge, Cambridge, UK

**Keywords:** microfabricated culture device, multiplexing, stem cell culture, bioprocess microfluidics

## Abstract

We present a multiplexed platform for a microfabricated stem cell culture device. The modular platform contains all the components to control stem cell culture conditions in an automated fashion. It does not require an incubator during perfusion culture and can be mounted on the stage of an inverted fluorescence microscope for high-frequency imaging of stem cell cultures. A pressure-driven pump provides control over the medium flow rate and offers switching of the flow rates. Flow rates of the pump are characterized for different pressure settings, and a linear correlation between the applied pressure and the flow rate in the cell culture devices is shown. In addition, the pump operates with two culture medium reservoirs, thus enabling the switching of the culture medium on-the-fly during a cell culture experiment. Also, with our platform, the culture medium reservoirs are cooled to prevent medium degradation during long-term experiments. Media temperature is then adjusted to a higher controlled temperature before entering the microfabricated cell culture device. Furthermore, the temperature is regulated in the microfabricated culture devices themselves. Preliminary culture experiments are demonstrated using mouse embryonic stem cells.

## Introduction

Pluripotent embryonic stem cells can be propagated indefinitely and differentiated into most adult cell types and thus potentially offer an unlimited supply of clinically relevant cells for cell-based therapies and drug discovery.^1–4^ To successfully manufacture the desired cell type in significant quantities and in a highly reproducible fashion, processes must be developed that precisely control the microenvironment in which cells are first expanded and then differentiated.^5–7^ In this microenvironment, numerous biological, physical, and chemical factors synergistically combine to control stem cell fate.^8^ Therefore, a large number of multivariable experiments will be needed to precisely define the optimum culture conditions.

Various input parameters such as medium composition, medium exchange rates, and temperature affect the proliferation and differentiation of embryonic stem (ES) cells. Structured and data-rich process development requires monitoring and control of these parameters along with monitoring of output parameters indicative of productivity and selectivity. A tight control over the microenvironment, including automated fluid handling, is required to achieve the necessary control over input parameters while time-lapse imaging of cultures provides a noninvasive and data-rich measure of process outputs. An automated microfluidic system with a time-lapse imaging system would therefore be of great benefit in this endeavor, with the combination of microfluidics with phase contrast and fluorescence microscopy enabling automated analysis of experimental outcomes. For example, such a system will allow the study of phenotype variations under different culture parameters such as flow modes, hydrodynamic shear stress, or oxygen tension levels with minimum of effort and resources. Attempts to address this and the fine control of the cellular microenvironment by microfluidic devices have been reported, for example, for mouse and human ES cells,^9–11^ for the study of regenerative processes,^12^ and for drug discovery applications.^13^

Integration of real-time optical monitoring with microfluidic culture devices remains challenging, particularly for long-term culture.^14^ Plate reader compatible, microfluidic culture systems have been recently reported to measure fluorescent tags.^15^ However, in such configurations, measurements can only be taken at discrete time points. A charged-coupled device (CCD) has been directly mounted to a microfluidic cell culture device to count cells, but this solution may not be suitable for high-resolution imaging of cell morphology or fluorescent labels.^16^ An inverted microscope remains attractive when high-quality images of cells are required for image analysis of phenotype or for determining cell numbers.

The advantages of using time-lapse imaging to characterize cells or entire organisms have been shown, for example, to monitor the development of zebrafish embryos,^17^ cellular metabolites, and ions using genetically encoded biosensors^18^ or to characterize induced pluripotent stem (iPS) cells.^19^ A microfluidic culture chamber on an inverted microscope has been previously implemented with a transparent heater to monitor the proliferation of HeLa cells and to maintain culture temperature for the duration of inspection on the microscope,^20^ and parallelization has recently been shown.^21^ Albrecht et al.^22^ have presented a microfluidic platform with time-lapse imaging in a 96-well format for mouse embryonic stem cells for the duration of 5 days. However, these systems focus on the investigation of specific parameters for small populations of cells, in conditions that differ significantly from traditional culture systems.

We previously presented the perfusion culture of human embryonic stem cells (hESCs) in co-culture in a microfabricated culture device.^23^ The device included a resealable chamber that facilitated the use of standard culture protocols, including the static seeding typically employed at laboratory scale. Although the device was designed to enable cell culture imaging with an inverted microscope, the device had to be placed in an incubator for temperature control. Therefore, images were obtained only at discrete time points and required the transport of the device. In addition, such transport results in a loss of control over culture conditions, which can have a significant impact on the reproducibility of embryonic stem cell cultures.^24^

As a further limitation, flow control was achieved with a syringe pump external to the incubator. With this pumping system, it was not possible to keep culture medium at appropriate storage temperatures. Furthermore, syringe pumps are typically prone to pulsatile flow, particularly with larger syringe volumes and lower flow rates. Both of these factors prevented longer term culture experiments.

In this contribution, we present a multiplexed platform for this device, which no longer requires an incubator during perfusion culture. We describe the integration of media handling and automation of the modular platform to facilitate high-frequency imaging of stem cell cultures. With our platform, the culture medium is stored in cooled reservoirs to prevent culture medium degradation during long-term experiments. Media temperature is controlled in the storage container and adjusted to a higher controlled temperature before entering the microfabricated culture device. Furthermore, the temperature is regulated in the microfabricated culture devices themselves. Finally, the platform implements control over medium flow rate and allows switching between two different media bottles in one experiment.

## Materials and Methods

### Fabrication of the Microfabricated Cell Culture Device with Culture Chamber Heating

The microfabricated culture devices were fabricated as previously reported^23^ except for the bonding of the poly(dimethylsiloxane) (PDMS) parts. The gasket and the microfluidic chip were bonded using an air plasma (PDC-002; Harrick Plasma, Ithaca, NY), and this assembly was then bonded to a glass slide using an air plasma. The bottom surface of the glass slide was coated with indium tin oxide (ITO) (576352; Sigma-Aldrich, Gillingham, UK) to allow resistive heating of the culture chamber.

The interface plate or top plate^23^ was made from a 5-mm-thick polycarbonate (PC) plate. The interface plate was fitted with four bores for M3 screws to keep the microfabricated cell culture device in place when mounted on the microscope. A pocket was milled into the interface plate to fit a thermocouple. For temperature monitoring, a pocket was milled (1.6 × 1.6 × 22.8 mm) to insert a negative temperature coefficient (NTC) thermistor (B57861S103F40; Epcos, Heidenheim, Germany). The thermistor was held in place using an M3 brass screw. The thermistor was in direct contact with the cell growth surface plane of the ITO microscope slide.

To electrically contact the ITO coating from the top of the device, two M3 threads for M3 × 30-mm standard brass screws (Clerkenwell Screws, London, UK) were manually cut into the interface plate. The two screws pressed against a copper strip on the top of the microscope glass slide. The copper strip was wrapped around the edges of the microscope glass slide and thus provided electrical contact from the top surface of the slide to the ITO coating on the bottom surface.

The bottom frame was made from aluminum (Al) and had one large opening (49.5 mm long, 26 mm wide) to facilitate observation of the culture chamber and the microfluidic chip. The area of the frame, which mechanically supported the microscope slide, was covered with Kapton tape (5413, 3/4 inch; 3M, St. Paul, MN) to electrically insulate the bottom aluminum frame from the ITO microscope slide.

The interconnect sockets at the inlet and outlet were fitted with Luer-Lock adapters (P-686; Upchurch, Oak Harbor, WA, USA) to quickly connect with tubing. At the inlet socket of the microfabricated cell culture device, an autoclavable three-way valve (PMMM-700-156W; Fisher Scientific, Loughborough, UK) was attached to this Luer-Lock adapter.

### Pressure-Driven Pump

Two 125-mL straight-sided polycarbonate wide-mouth jars (2116-0125; Nalgene, Rochester, NY) were each fitted with three ports: an inlet, an outlet, and a pressure-relief port. Inlet and outlet ports included a pipe made out of stainless steel, press-fitted to the ports. Inlet and pressure-relief port each included a syringe filter (Minisart 16596; Sartorius Stedim, Goettingen, Germany) to maintain sterility. The jars were pressurized using an external gas feed applied at the inlet to drive flow. The gas flow rate was controlled via a pressure regulator (ITV0011-2BL-Q; SMC, Milton Keynes, UK), while the flow path was controlled by four three-way valves (S070B-6CC; SMC). Characterization of flow rate versus pressure was performed with DI water using a thermal mass-based flow meter (SLG1430-480; Sensirion, Staefa, Switzerland). Pressure regulator outputs 1 bar at 5 volts direct current (VDC).

### Medium Reservoir Cooling

Both jars of the pressure-driven pump were immersed in a water bath. The water bath consisted of an aluminum box with appropriate openings for the jar. A Peltier element with a heat sink and cooling fan (AA-60-12-00-00; Supercool, Gothenburg, Sweden) and a negative temperature coefficient (NTC) thermistor (B57703M103G; Epcos) were attached inside of the aluminum box.

### Preheater

The preheater was formed of two parts made out of poly(methylmethacrylate) (PMMA). One contained a milled groove (1.6 × 1.6 mm) and an NTC thermistor (B57045K103K; Epcos); the other embedded a resistive heating foil (HK5160R157L12B). When the two parts were clamped together, the groove held the tubing (for the culture media provision) in place and brought the resistive heater and thermistor in contact with each other.

### Bubble Trap

The bubble trap consisted essentially of a T-junction made of a perpendicular bore that aligned and connected with a horizontal bore. The horizontal bore connected to tubing upstream and downstream via standard fittings. When an air bubble entered the bubble trap, it passed through the T-junction and thus rose in the perpendicular bore, which removed it from the culture medium (see **Suppl. Fig. S1** for a technical drawing).

### Closed-Feedback Loops for Temperature Control

Closed-feedback control of the temperature of the water bath, the preheater, and the culture devices was achieved using the same pulse-width modulation (PWM) control loop (see **Suppl. Fig. S2**). The digital output of a data acquisition (DAQ) card (USB-6221; National Instruments, Austin, TX) controlled a solid-state relay (CMX60D10; Crydom, San Diego, CA) to switch on/off the current from a power supply. The signal from the NTC thermistor was read via a circuit into the DAQ card. The algorithm for the PWM was programmed using LabVIEW (National Instruments, Austin, TX).

### Platform Housing

All components of the platform were housed in two modules, a media-handling module and a microscope module. The microscope module was assembled within two enclosures attached to each other. The main enclosure (60113234; Fibox, Stockton on Tees, UK) housed the three microfabricated cell culture devices, the flow splitter, the preheater, the bubble trap, and waste vials to collect the spent medium from the cell culture devices. A second enclosure (PCM 175/150 G; Fibox) housed all electrical circuits to control the temperature of the ITO microscope slides (of the microfabricated cell culture devices) and of the preheater and accommodated the relay that controlled the valve to switch the flow between the two media reservoirs. The microscope module, a third enclosure (CAB PC 303018 G; Fibox), was attached with M3 screws to the stage of an inverted fluorescence microscope. The media-handling module contained the pressure-driven pump with two culture medium reservoirs, which were immersed in a water bath to store the culture medium at a low temperature. In addition, this module accommodated the control elements and circuitry for the valves and for the temperature control of the water bath.

### Power Supplies

Various power supplies were required for operation—that is, for the ITO-based heating of the culture devices (IPS 2303; Iso-Tech, Southport, UK), the Peltier cooling element (IPS 2303; Iso-Tech), the fan of the Peltier element (UG01B; Maplin, UK), the valves of the pressure-driven pump system and the preheater (L48BQ, Maplin, Rotherham; LT30-2, Farnell Instruments, Leeds, UK), and the pressure regulator (EP-920; Eagle Technology, Cape Town, South Africa). These were not included in the platform housing.

### Cell Maintenance

Mouse embryonic stem cells (mESCs) with an Oct-4-GiP reporter were obtained from Stem Cell Sciences (Edinburgh, UK). mESCs (< passage 40) were routinely cultured in T25 flasks (136196; Nunc, Roskilde, Denmark) coated with 0.1% (w/v) filtered water porcine gelatin (G1890; Sigma) and kept in a humidified incubator at 37 °C and 5% CO_2_. In total, 6 mL of medium per flask was exchanged daily. Medium consisted of Glasgow Minimal Essential Medium (GMEM) (G5154; Sigma) and was supplemented with 10% (v/v) fetal bovine serum (FBS) (EU-000-F; Sera Laboratories International, West Sussex, UK), 1% (v/v) glutamax (35050-038; Invitrogen, Carlsbad, CA), 1% (v/v) sodium pyruvate (11360-039; Invitrogen), 0.2% (v/v) of 2-β-mercaptoethanol (31350; Invitrogen), and 1% (v/v) of antibiotic-antimycotic solution (15240-062; Invitrogen). Following filter sterilization, 500 µL of leukemia inhibitor factor (LIF) (ESG1107; Millipore, Watford, UK) was added to the medium.

Cells were passaged every 2 days. New flasks were incubated with 3 mL of 0.1% (w/v) porcine gelatin solution for at least 10 min prior to passaging in a laminar flow hood at room temperature. Cells were washed first with approximately 3 mL of Dulbecco’s phosphate-buffered saline (DPBS). DPBS was aspirated and 500 µL of trypsin added to the cells in the flask and incubated at 37 °C for 3 min. To quench trypsin, approximately 4.5 mL of complete medium was added to a flask. The cell suspension was aspirated and added to a 50-mL centrifuge tube (210 261, CellStar Tubes; Greiner Bio-One, Monroe, NC). Cells were spun in a centrifuge (Centrifuge 5810R; Eppendorf, Hamburg, Germany) at 1200 rpm for 3 min at room temperature. The supernatant was aspirated and the pellet resuspended in fresh medium. Cells were split in ratios of 1 to 10.

### Cell Culture

The microfabricated culture devices were assembled without the lid, placed in a glass Petri dish, and autoclaved with the dish. To sterilize tubing, both ends of the tubing (the end that connects to the fluid outlet of the medium reservoirs and the other end that connects with the microfabricated devices) were fitted with syringe air filters. Tubing was then filled with ethanol or isopropanol and left in the tubing for at least 30 min. Tubing was subsequently rinsed at least twice with sterile DPBS. Laboratory pipettes with 200-µL tips were used for coating and cell seeding steps. The glass surface of the culture chamber was coated with 0.1% (w/v) porcine gelatin solution and incubated at room temperature. Cells were passaged as for routine maintenance. The concentration of the resuspended cells was measured using a hemocytometer and adjusted to achieve a seeding density of approximately 10,000 cells cm^−2^. To minimize culture medium evaporation during the time cells attached to the bottom of the culture chamber, the devices were placed in a closed Petri dish with a cap filled with 3 mL of sterile DPBS before moving them to the incubator for 24 h. Meanwhile, the reservoirs, the lids of the microfabricated devices, and the screws were then autoclaved. After 1 day of incubation, mESCs attached to the gelatin-coated glass slides and all microfabricated cell culture devices were closed and primed with culture medium. After cell seeding in the microfabricated devices, the microscope module was placed in a laminar flow hood and all three microfabricated devices inserted and secured inside the module. The reservoirs were filled with 50 mL of culture medium. The filters on both ends of the tubing were removed and tubing attached to the microfabricated devices and the fluid outlet ports of the culture medium reservoirs. The microscope module and the two reservoirs (with the filters to maintain sterile conditions still attached to the inlet and purge ports) were transferred to the stage of an inverted microscope. After the transfer, the modules were connected to the computer and the power supplies. The gas lines of the media-handling module were attached to the filters of the inlet and purge ports of the reservoirs. Culture medium tubing from the reservoirs to the microfabricated culture device was then primed using a combination of manual valves fitted to the bubble trap and the inlet ports of the microfabricated device. After priming, these manual valves were closed, and the LabVIEW routine started. Medium was then perfused at 300 µL/h^−1^. A compressed air laboratory outlet was used as the external gas feed for the preliminary cell culture experiments but could be replaced with a gas mix bottle consisting of 5% CO_2_ balanced air for CO_2_ control. Phase-contrast images (10× magnification) were taken using an inverted fluorescence microscope (Eclipse TE2000-U; Nikon, Tokyo, Japan), equipped with a camera (Scout scA1400; Basler Vision Technologies, Ahrensburg, Germany).

## Results and Discussion

### Microfabricated Cell Culture Device with Culture Chamber Heating

**Figure 1.**
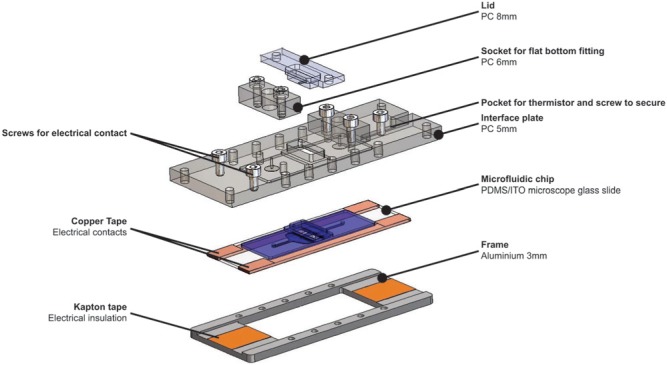
Exploded view of the microfabricated cell culture device. PC, polycarbonate.

The microfabricated culture device is based on the modular design used for the perfusion of hESCs^23^ (**Fig. 1**). Briefly, the device in this work consisted of the following components: a microfluidic chip made from PDMS supported by an ITO-coated glass slide, a (top) interface plate made from PC and a bottom frame made from aluminum (Al), two interconnect sockets (PC), and a lid (PC) to reversibly seal the culture chamber of the device.

The main modifications to the previous device to adapt it for use on an inverted microscope included the glass microscope slide, which replaced the tissue culture polystyrene slide (TC-PS). The glass slide with its ITO coating on the bottom surface of the slide facilitated localized heating of the culture chamber. Additional changes had to be made to the (top) interface plate and bottom frame to incorporate a thermistor for temperature monitoring and to enable electrical contacting of the ITO coating from the top of the device. Furthermore, low-profile interconnect sockets were implemented to ensure that the devices would fit in the space between the stage and the condenser of the microscope.

**Figure 2.**
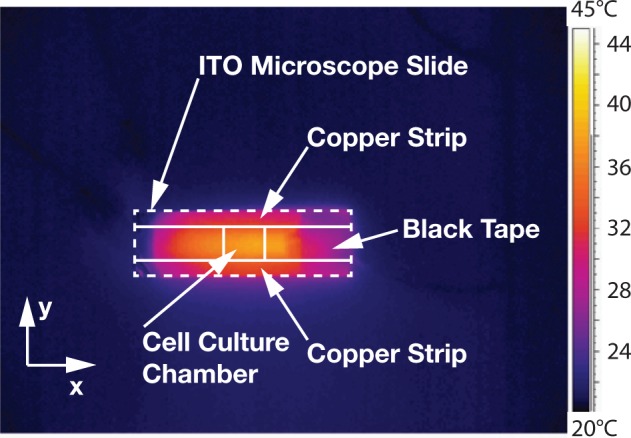
Infrared image of an indium tin oxide (ITO) microscope slide used as a heater.

Based on the temperature uniformity of ITO microscope slides as reported by Lin et al.,^25^ we designed the device such that the thermistor was positioned outside of the PDMS microfluidic chip (i.e., at a distance from the culture chamber). For a preliminary assessment of the spatial distribution of the temperature, we used a thermal imaging handheld camera (ThermaCAM SC360; FLIR Systems, Täby, Sweden) and visualized the infrared signature of an ITO microscope slide (**Fig. 2**). Images were analyzed using ImageJ (version 1.43; National Institutes of Health, Bethesda, MD). Temperature distribution was analyzed in particular for the culture chamber. For each direction, three measurements were taken and the mean and the relative standard deviations calculated. In the *x*- and *y*-direction, the temperatures deviated approximately by 7% and 5%, respectively.

### Design and Assembly of the Multiplexed Platform

**Figure 3.**
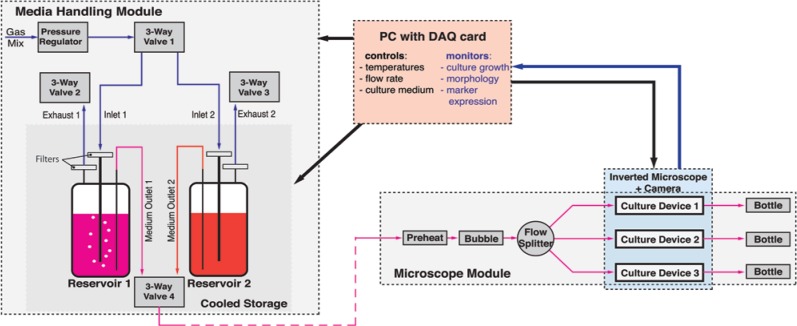
Schematic of the multiplexed platform. The platform consists of two modules: the microscope module and the media-handling module. The media-handling module provides cooled storage of culture medium in two reservoirs and a pressure-driven pump (consisting of a set of valves and a pressure regulator) to pump the culture medium from either of the two reservoirs. In the microscope module, medium is preheated before air bubbles are removed in a bubble trap, and culture medium flow is split into three cell culture devices. A LabVIEW routine is used to control all important culture parameters such as the different temperatures, selection of the reservoir for culture medium pumping, and flow rate.

**Figure 4.**
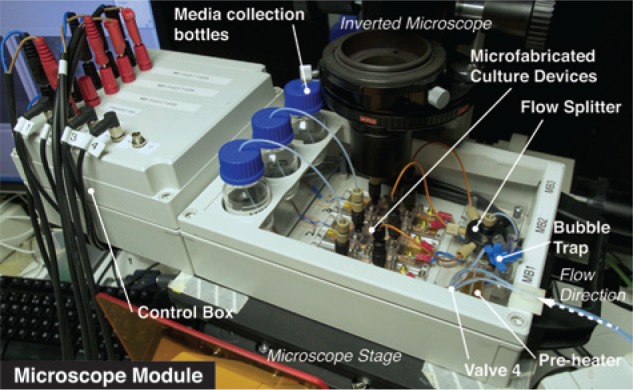
Photograph of the microscope module attached to the stage of an inverted fluorescence microscope.

The multiplexed platform consisted of two modules, a media-handling module and a microscope module (**Figs. 3** and **4**). The microscope module housed the three microfabricated cell culture devices, the flow splitter, the preheater, the bubble trap, and vials to collect the spent medium (waste) from the cell culture devices. It included also all electrical components for the operation of the platform. The media-handling module (see **Suppl. Fig. S3** for a photograph) contained the pressure-driven pump with two culture medium reservoirs, which were immersed in a water bath to store the culture medium at a low temperature, to prevent the degradation of culture medium during multiday experiments (and thus permitting long perfusion times without having to replace empty reservoirs with new full ones). In addition, this module accommodated the control elements and circuitry for the valves and for the temperature control of the water bath.

For operation, a three-way valve (valve 1 in **Fig. 3**) directed the gas from an external source to the desired culture medium. Use of an external gas source allows flexibility in gas composition and, for example, could be a cylinder containing 5% CO_2_ as found in incubator atmospheres. A stainless steel tube immersed in the reservoir sparged the gas into the culture medium. The pressure in the headspace of the reservoir pumped culture medium out of the reservoir and into the microscope module via a further three-way valve (valve 4). The pressure regulator thus determined the pressure in the reservoirs, which in turn determined the flow rate in the microscope module. By switching the three-way valve 1, either the left or the right reservoir acted as the pump, which effectively allowed switching between two culture media. This could be employed, for example, to initially expand stem cells in the microfabricated devices and then switch to a different culture medium for stem cell differentiation. The volume of the two autoclavable reservoirs was sufficient to sustain long-term continuous perfusion of the three culture devices. To release any excess pressure from the reservoirs, both reservoirs had a filtered gas exhaust port, which was opened using valve 2 or 3, respectively. This feature also enabled the rapid release of any pressure downstream of the reservoirs, to swiftly halt the flow in the microfabricated devices. As the cold culture medium entered the microscope module, it was first preheated to bring its temperature closer to cultivation temperature. The culture medium then passed through a bubble-trap to remove any remaining gas bubbles. For multiplexing, the flow was split after the bubble trap into three streams and—after perfusing the cells cultured in the three devices—spent media were collected in small bottles for each device independently. These could easily be removed from the microscope module for metabolite analysis.

By assigning the functionalities necessary for operation over several components with individual functions, each of these components can be developed independently. Optimization of individual components, or expansion of the platform with additional functionalities, is therefore greatly facilitated; a complete redesign or remanufacture of the entire microfluidic platform is thus not required. Furthermore, exchange or disposal of used or defect parts, respectively, is facilitated. A drawback of this modularity is that each component has its dead volumes. In addition, tubing is necessary to connect the components to each other. This increases the total volume upstream of the culture devices and thus increases the switching times (i.e., the time until a change of culture medium reservoir effectively changes the culture medium in the microfabricated device). In principle, such dead volumes could be reduced or minimized by recombining different functions into one device, once the development and optimization of the platform have been completed.

Operation of the multiplexed platform was automated using a LabVIEW routine, which controlled all components of the platform. For the automation routine to control multiple pieces of equipment with each executing at different time points and different rates, the issue of race condition had to be addressed. *Race condition* refers to the situation when there are two (or more) signals that can influence a single output, with the output then being dependent on which signal reaches the output first. In our routine, this would, for example, have an impact on the logging of output data from several pieces of equipment, which sample the readings at different time points. This problem was overcome by creating individual subroutines that executed specific tasks. Each of these subroutines passed its readings to a main program, which recorded the data at fixed intervals. This modular approach was also beneficial during routine development, for example, to debug the code.

### Characterization of the Temperature Control

**Figure 5.**
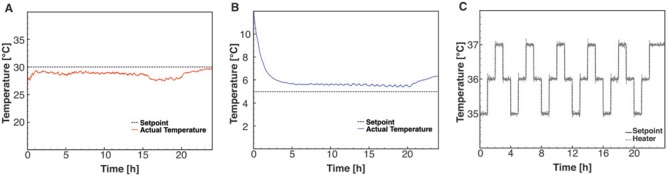
Temperature control for the various heaters. (**A**) Time course data of the measured temperature in the preheater. Temperature above 27 °C is maintained for 24 h. (**B**) Time course data of the Peltier cooling element of the water bath. A temperature of 6 °C is achieved within 5 h and maintained for 15 h. (**C**) Time course data of the temperature control in one of the three cell culture devices. The temperature of each cell culture device is varied between 35 °C and 37 °C in steps of 1 °C and maintained at the set temperature for 1 h.

A closed-loop feedback control method was developed to control the temperature of the microfabricated device, the water bath, and the preheater. The feedback loop used a thermistor, a software-based PID algorithm and PWM (**Suppl. Fig. S2**). For the preheater, which was designed to heat the culture medium from the cooled reservoirs to a temperature near the ideal cultivation temperature, a temperature between 27 °C and 28 °C was achieved within 2 h (**Fig. 5A**). Given the low flow rates, the culture medium will be heated further in the microfabricated cell culture device before it enters the culture chamber. However, to avoid the formation of bubbles after the bubble trap, it will be necessary to achieve temperatures closer to the cultivation temperature. This may require a more powerful heater, which would possibly also improve the temperature stability. For the cooling plate, a temperature of 6 °C was achieved within 5 h (**Fig. 5B**). It is expected that this will allow storage of the culture medium in conditions suitable for long-term operation.

To characterize the closed-loop control of the ITO glass heating of the microfabricated device, we investigated with what precision the three temperatures (35 °C, 36 °C, and 37 °C) were maintained over time and the reproducibility when switching between the temperatures. As can be seen from **Figure 5C**, the closed-loop control was capable of maintaining a temperature within ±0.2 °C maximum deviation from the set point. In addition, switching between the three temperatures several times over 24 h was highly reproducible. However, to ensure that the cells experience the desired temperature, further characterization will be necessary. These include analysis of differences between the temperature measured by the thermistor compared with the temperature in the culture chamber, temperature gradients within the culture chamber, and characterization of the robustness of the temperature control over longer periods for long-term cultivations.

### Characterization of the Pressure-Driven Pump

**Figure 6.**
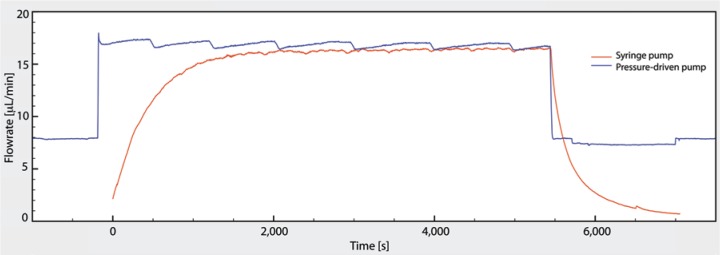
Comparison of flow rates generated in a syringe pump and our pressure-driven pump. Both pumps were turned on, left running for an hour, and then stopped. The red line depicts the temporal course of the flow rate generated by the syringe pump, while the blue line represents the temporal course of the flow rate generated by our pressure-driven pump.

To characterize the pressure-driven pump, we first investigated its response time and compared the transients of our pressure-driven pump with a syringe drive (Model 100; KD Scientific, Holliston, MA) (**Fig. 6**). The flow rates were measured with a nano flow sensor whose capillary had an inner diameter of 480 µm. Flow generated with the syringe pump drive led to long transient times of about 15 to 30 min until flow stabilized. Long transient times when using syringe pumps have been reported previously,^26^ although the exact time constants depend on the specifics of the setup such as the use of plastic syringes (instead of glass syringes), inner diameter of the syringes compared with the diameter of the tubing, the backpressure from the flow sensor and tubing, and elasticity of the tubing. In comparison, the transients with our pressure-driven pump were very short. Therefore, with our pump, on/off flow conditions can be created with good temporal control over the flow rates (see **Suppl. Fig. S4** for further validation and switching of flow rates). Exposure of the cells to shear stresses, for example, is clearly temporally confined. In addition, cell-signaling factors do not continue to be washed out over an undefined transient period—that is, when desired, static culture conditions can be achieved almost instantaneously. Likewise, culture perfusion (i.e., the “blank slate”–like delivery of growth factors for the cells) can be started at the desired flow rate without long transients.

A characteristic observed with all flow rate measurements was the “sawtooth”-like time profile of the flow rate. This was due to the way the pressure regulator of the pump controlled its output pressure. As culture medium was pumped out of the reservoirs, the gas volume between pressure regulator and culture medium surface in the reservoirs increased and pressure dropped. As soon as the pressure fell outside the range, fast-switching solenoids in the regulator opened the pneumatic connection with the supply pressure to increase the output pressure. A finer control of the pressure (e.g., by using a pressure regulator with a narrower pressure range) would result in smaller variations of the flow rate.

To characterize the flow rates in the multiplexed platform, flow rates were measured at different locations. First, the flow rates at each port of the flow splitter manifold were measured for three different pressure regulator settings. The mean and the standard deviation of the flow rates over 30 min for each port and pressure setting are summarized in **Table 1**. The coefficient of variation (CV) or relative standard deviation (the ratio of the standard deviation to the mean) of the flow rates was between 2% and 6%. The CV of the means was 6% for the pressure settings of 300 mV and 500 mV and 3.5% at 400 mV (the pressure regulator outputs 1 bar at 5 VDC).

**Table 1.** Flow Rates Measured at Each of the Three Flow Splitter Ports for Different Pressures.

Regulator Setting, mV	Port 1, µL/min	Port 2, µL/min	Port 3, µL/min
300	9.2 (0.3)	8.7 (0.4)	8.2 (0.5)
400	15.1 (0.5)	16.1 (0.3)	15.9 (0.6)
500	20.7 (1.1)	23.2 (0.5)	21.5 (0.5)

Pressure regulator outputs 1 bar at 5 volts direct current. Flow rates were measured at each port while the other two ports were sealed. Average and standard deviation (in parentheses) from three measurements (*n* = 3) are reported.

Second, the flow rate was measured at one port of the flow splitter manifold and the pumping switched between the culture medium reservoirs. The average and the standard deviation for each port and pressure setting are summarized in **Table 2**. For all three pressure settings, the switch from reservoir 1 to reservoir 2 increased the flow rate, while the return to reservoir 1 decreased the flow rates. The change in flow rate between the two reservoirs was significant for all three settings. The flow rates obtained by pumping from reservoir 1 before and after the switch to reservoir 2 were comparable at the higher flow rates—a CV of 2.7% and 3.6% for the 400-mV and 500-mV settings, respectively. At the lowest flow rate with the 300-mV setting, a CV of 9.5% was calculated. Furthermore, the flow rates were in a similar range to those from **Table 1**.

**Table 2.** Flow Rates Measured at the Same Flow Splitter Port When Switching between Culture Medium Reservoirs.

Regulator Setting, mV	Reservoir 1, µL/min	Reservoir 2, µL/min	Reservoir 3, µL/min
300	8.9 (0.3)	9.3 (0.3)	8.0 (0.3)
400	14.6 (1.4)	15.9 (0.9)	14.2 (0.3)
500	21.0 (2)	24.2 (0.4)	20.3 (1.34)

Average and standard deviation (in parentheses) from three measurements (*n* = 3) are reported.

Third, the flow rates after the cell culture devices were measured. In **Table 3**, the culture device numbering matches with the port numbering from **Table 1**. At the lowest pressure setting, the flow rates were not consistent for the different devices. For the higher flow rates, the differences were within 10% to the flow rate at the corresponding ports.

**Table 3.** Flow Rates Measured at the Outlet of Each Microfabricated Cell Culture Device at the Same Port.

Regulator Setting, mV	Device 1, µL/min	Device 2, µL/min	Device 3, µL/min
300	9.9 (0.3)	1.2 (0.1)	3.5 (0.7)
400	16.4 (0.4)	15.1 (0.4)	13.3 (1.2)
500	23.6 (0.4)	22.6 (0.3)	20.3 (1.1)

Average and standard deviation (in parentheses) from three measurements (*n* = 3) are reported.

**Figure 7.**
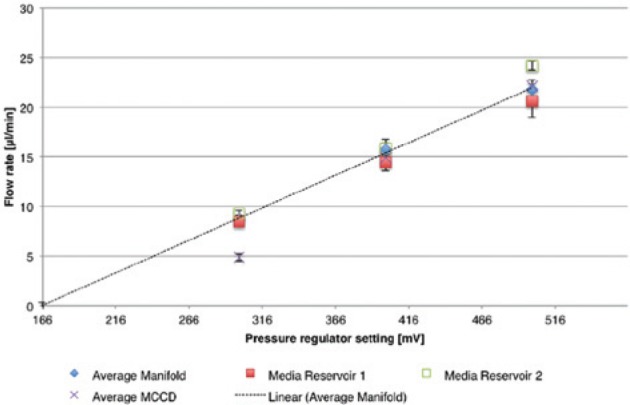
Flow rate compared with pressure regulator settings. The average flow rates measured after the manifold for each port, after each cell culture device, and for both media reservoirs for the three pressure regulator settings (300, 400, and 500 mV) are plotted. A linear curve fit of the flow rates measured after the manifold (*R* ^2^ = 0.998) can be used to predict the flow rate due to the linear correlation of pressure and flow rate. MCCD, microfabricated cell culture device.

Finally, all flow rates were plotted against the pressure regulator settings in one graph (**Fig. 7**). The flow rates from the three ports of the flow splitter manifold and for each cell culture device were averaged. The mean flow rate of the flow splitter showed a reasonably linear correlation with the applied pressure. A minimum pressure is required to obtain a flow rate higher than zero. This is expected given the pressure resistance of the tubing and flow splitter manifold. The linearity of the correlation in principle allows determining a flow rate for the operation of the multiplexed platform directly from pressure settings without the need of flow rate detection. Since the characterization of the pump is performed with water, a recalibration of the pump would be necessary when using culture medium with properties that differ significantly from water.

### Platform Assembly and Setup for Cell Culture

**Figure 8.**
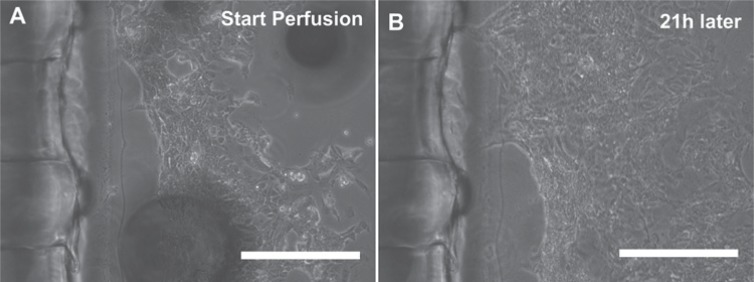
Phase-contrast microscope images (10×; scale bar = 200 µm) of mouse embryonic stem cells in the cell culture device (**A**) at the beginning and (**B**) after 21 h of continuous culture medium perfusion.

To demonstrate assembly and setup of the multiplexed platform for cell culture, we performed a preliminary culture experiment with mESCs. A compressed air laboratory outlet was used as the external gas feed for the preliminary cell culture experiments but could be replaced with a gas mix bottle consisting of 5% CO_2_ balanced air for CO_2_ control. Under continuous perfusion for 24 h at 300 µL/h^−1^, the cells started to proliferate, as shown in **Figure 8**. After 21 h, the cells appeared viable, and there was no sign of cell apoptosis. Furthermore, we did not observe any visible contamination.

In conclusion, we have developed a multiplexed platform for a microfabricated culture device, with which embryonic stem cell culture previously had been successfully performed.^23^ The platform provides the features of an incubator and fits onto an inverted fluorescence microscope and will thus in the future permit time-lapsed imaging of stem cell cultures. Temperature is controlled at several places of the multiplexed platform, to maintain culture medium at a low temperature to minimize medium degradation and to control the on-chip temperature during culture. The platform contains a pressure-driven pump, which offers rapid switching of flow rates (including stopping of the flow) to control the washout of culture medium, including the cell-secreted factors. By altering the inlet gas type, different gas tensions can be applied to the inlet stream of the culture devices.

The flow control and multiplexing afforded by this platform will in the future allow the parallel operation of three culture devices. Starting from the same preculture (i.e., the same passage number) is crucial to assess reproducibility of a stem cell culture protocol. Using the previously demonstrated direct seeding,^23^ it will become possible to split a preculture into the devices of our platform and analyze the reproducibility from very similar starting conditions. This, for example, can be exploited to assess the impact of different device geometries. Furthermore, the platform will also allow direct comparison of different culture variables for stem cells. These include the temperature, the type of cells, different extracellular matrices, and different medium exchange regimes, all from the same passage number. We are currently working on image-processing algorithms to rapidly quantify the confluency of adherent stem cell cultures and the integration of oxygen sensors. This will provide relevant information of the culture growth kinetics in real time and thus offer to correlate the culture conditions with cellular behavior for stem cells.
